# Supplementary material for: The Application of Bilayer Heterogeneous MOFs in pH and Heat-Triggered Systems for Controllable Fragrance Release
Source: Materials (Basel). 2024 Mar 12;17(6):1310. doi: 10.3390/ma17061310 (PMC10972472; doi:10.3390/ma17061310)
Supplement: Supplementary file 1 [file materials-17-01310-s001.zip › materials-2891493-supplementary.pdf]

materials

Supplementary Materials

Table S1. Comparison of encapsulation methods for different functional materials.

Methods	Materials	Performances	Characteristics	Ref.
Spray drying	Chitosan Encapsulated Orange Oil	90% encapsulation efficiency	Wide range of applications and easy to scale up, but limited by high temperatures	[1]
Chemical polymerization	Epoxy resin encapsulated capsaicin	93% release in ethanol	Fast reaction rate and gentle reaction process, but lower mechanical integrity of the capsule	[2]
Mesoporous adsorption	Mesoporous silica encapsulated curcumin	6.6% loading efficiency	Non-specific, but with low loading efficiency	[3]
**Multiple adsorption**	**Bilayer ZIF-8-on-ZIF-8 encapsulated vanillin**	**25.68% loading efficiency**	**Dual-responsive release system with pH and thermal trigger control and high load efficiency**	**This work**

Table S2. Full name corresponding to the abbreviation.

Abbreviations	Full name
MOFs	Metal-organic frameworks
ZIFs	Zeolitic imidazolate frameworks
2-MIm	2-methylimidazole
M	ZIF-8
MM	ZIF-8-on-ZIF-8
VM	Vanillin@ZIF-8
VMM	Vanillin@ZIF-8-on-ZIF-8
VMVM	Vanillin@ZIF-8-on-vanillin@ZIF-8
GGA	Generalized gradient approximation
PBE	Perdew-Burke-Ernzerhof
PAW	Projector augmented-wave
TAC	Total adsorption capacity
ISAC	Inner surface adsorption capacity
V@M	ZIF-8 adsorbed vanillin
V@MM_2	ZIF-8-on-ZIF-8 (1:2) adsorbed vanillin
V@VM	Vanillin@ZIF-8 adsorbed vanillin
V@VMM	Vanillin@ZIF-8 adsorbed vanillin
XRD	X-ray diffraction
SEM	Scanning electron microscope
TEM	Transmission electron microscopy
HRTEM	High-resolution transmission electron microscopy
RT	Room temperature

Table S3. Comparison of single-layer ZIF-8 and double-layer ZIF-8-on-ZIF-8 performance.

Samples	ISAC	TAC	CR (pH=3, 10 h)	CR (60 °C, 48 h)
ZIF-8 (M)	6.17%	19.97%	91 wt%	90 wt%
ZIF-8-on-ZIF-8 (MM)	10.89%	25.68%	76 wt%	69 wt%
vanillin@ZIF-8 (VM)	9.80%	24.87%	91 wt%	88 wt%
vanillin@ZIF-8-on-ZIF-8 (VMM)	11.26%	30.09%	67 wt%	69 wt%

Table S4. Secondary kinetic fitting parameters for different samples at different pHs.

Samples		pH			
		3	5	6.5	7
M	q_e	97.685	85.751	68.800	58.000
	k	0.016	0.011	0.012	0.017
	R^2	0.987	0.996	0.989	0.982
MM	q_e	108.688	77.363	56.534	46.541
	k	0.002	0.004	0.011	0.013
	R^2	0.960	0.968	0.982	0.982
VM	q_e	99.222	85.068	57.575	49.575
	k	0.011	0.013	0.022	0.012
	R^2	0.999	0.998	0.981	0.989
VMM	q_e	77.574	66.354	56.526	49.332
	k	0.008	0.010	0.010	0.011
	R^2	0.995	0.991	0.983	0.985

Table S5. Comparison of encapsulation properties of functional materials with different matrixes.

Matrixes	Functional materials	Methods	Loading capacity	Cumulative release	Ref.
ZIF-8	Physcion	Adsorption	11.49 wt%	88.72 wt% (pH=5, 72 h)	[4]
ZIF-8@gallic acid@Fe	5-fluorouracil	Adsorption	24.77 wt%	54 wt% (pH=5, 12 h)	[5]
ZIF-8	Gentiopicroside	Adsorption	10.77 wt%	81.31 wt% (pH=5, 50 h)	[6]
Whey protein isolate	vanillin	Non-thermal spray-freeze-drying	39.22 wt%	——	[7]
**ZIF-8**	**vanillin**	**Adsorption**	**19.97 wt%**	**84.42 wt% (pH=5, 48 h)**	**This work**
**ZIF-8-on-ZIF-8**	**vanillin**	**Adsorption**	**25.68 wt%**	**74.31 wt% (pH=5, 48 h)**	**This work**

Table S6. Secondary kinetic fitting parameters for different samples at different temperatures.

Samples		Temperature		
		-4 °C	RT	60 °C
M	q_e	55.524	58.862	81.420
	k	0.009	0.017	0.009
	R^2	0.991	0.983	0.933

MM	q_e	42.786	46.541	70.644
	k	0.011	0.013	0.005
	R^2	0.980	0.982	0.967
VM	q_e	30.685	49.579	95.295
	k	0.023	0.012	0.004
	R^2	0.940	0.989	0.988
VMM	q_e	35.994	49.330	65.376
	k	0.014	0.012	0.016
	R^2	0.971	0.985	0.959

References

1. Y. Li, L. Ai, W. Yokoyama, C.F. Shoemaker, D. Wei, J. Ma, F. Zhong, Properties of chitosan-microencapsulated orange oil prepared by spray-drying and its stability to detergents, Journal of Agricultural and Food Chemistry 61 (2013) 3311-3319.
2. T. Wang, X. Liu, J. Luo, G. Sun, R. Liu, Reduction of interfacial polycondensation self-limitation through solvent swelling to dynamically modulate shell thickness and structure of microcapsules, Polymer 285 (2023) 126369.
3. X. Sun, N. Wang, L. Yang, X. Ouyang, F. Huang, Folic acid and PEI modified mesoporous silica for targeted delivery of curcumin, Pharmaceutics 11 (2019) 430.
4. N.A. Soomro, Q. Wu, S.A. Amur, H. Liang, A.U. Rahman, Q. Yuan, Y. Wei, Natural drug physcion encapsulated zeolitic imidazolate framework, and their application as antimicrobial agent, Colloids and Surfaces B: Biointerfaces 182 (2019) 110364.
5. M. Ozsoy, V. Atiroglu, G.G. Eskiler, A. Atiroglu, G. Arabaci, M. Ozacar, Activities of gallic acid and Fe doped, and glucose oxidase or gold modified ZIF-8 based drug delivery systems in triple negative breast cancer, Journal of Drug Delivery Science and Technology 87 (2023) 104878.
6. S.A. Amur, N.A. Soomro, Q. Khuhro, Y. Wei, H. Liang, Q. Yuan, Encapsulation of natural drug gentiopicroside into zinc based Zeolitic Imidazolate Frameworks (ZIF-8): In-vitro drug release and improved antibacterial activity, Journal of Drug Delivery Science and Technology 84 (2023) 104530.
7. S.Y. Hundre, P. Karthik, C. Anandharamakrishnan, Effect of whey protein isolate and b-cyclodextrin wall systems on stability of microencapsulated vanillin by spray–freeze drying method, Food Chemistry 174 (2015) 16–24.
